# Disabled Nurses and Their Dependants

**Published:** 1920-03-06

**Authors:** 


### Disabled Nurses and their Dependants.

### Special Grants and Allowances.

The Special Grants Committee of the Ministry of Pensions have framed new regulations under which certain grants and allowances may be made in special cases to war disabled nurses and their dependants. These regulations, which take effect forthwith, apply to members of the Royal Naval Nursing Service and Reserve, Queen Alexandra's Imperial Nursing Service, the Army Nursing Service, the Territorial Force Nursing Service, and any other nursing service or reserve which is admissible under the Royal Warrant.

### Nurses.

A supplementary or special allowance may be granted to a nurse where, in consequence of serious disablement arising from service during the war, she is unable to maintain herself in her pre-war standard of comfort, but the supplementary or special allowance, together with any State pension which may be awarded, shall not exceed £90 a year. In exceptional cases grants may be made to nurses to meet temporary distress or emergency. Recoverable advances may be made to nurses pending the receipt of moneys due, or which the Special Grants Committee consider may become due, from the State in respect of pension, gratuity, or other payment, provided that such advances shall only be made in cases of real necessity.

### Dependants.

Where the parent, brother, or sister of a nurse is deprived by her death, in circumstances arising from service during the war, of any regular support which the parent, brother, or sister was receiving from the nurse before or during the war, or might reasonably have expected to receive from the nurse after the war, a supplementary or special allowance may be granted which, with any State pension which may have been awarded, shall not exceed the actual or prospective dependence, and shall not exceed the total of £60 a year.

### Children.

When a nurse, by reason of disablement arising from service during the war, is unable adequately to maintain her children, or where a nurse dies in consequence of war service, allowances for maintenance and education, not exceeding in amount those which may be paid under the Royal Warrant or the Special Grants Committee's Regulations for the maintenance and education of the children of a disabled or deceased officer, may be granted if the husband or widower of the nurse is deceased or if the Committee are satisfied that he cannot be expected to support the children.

Applications for allowances or grants should be headed " S. G. O.," and addressed to the Secretary, Special Grants Committee, Officers' Branch, Millbank House, Westminster, S.W.1.

